# Recent Knowledge in the Application of *Saccharomyces cerevisiae* in Aquaculture: A Bibliometric and Narrative Review

**DOI:** 10.3390/antibiotics14080736

**Published:** 2025-07-22

**Authors:** Elshafia Ali Hamid Mohammed, Béla Kovács, Károly Pál

**Affiliations:** 1 Department of Animal Husbandry, Institute of Animal Science, Biotechnology and Nature Conservation, Faculty of Agricultural and Food Sciences and Environmental Management, University of Debrecen, 4032 Debrecen, Hungary; 2 Doctoral School of Animal Science, University of Debrecen, 4032 Debrecen, Hungary; 3 Agricultural Research Corporation, Integrated Pest Management Research Center, Wad-Madani 21111, Sudan; 4 Institute of Food Sciences, Faculty of Agricultural and Food Sciences and Environmental Management, University of Debrecen, 4032 Debrecen, Hungary; kovacsb@agr.unideb.hu (B.K.); pal.karoly@agr.unideb.hu (K.P.)

**Keywords:** *Saccharomyces cerevisiae*, probiotics, synbiotics aquaculture, growth performance, disease resistance, bibliometric analysis

## Abstract

Aquaculture is a key food production sector responsible for meeting the nutritional needs of a rapidly growing global population. However, the emergence of disease outbreaks has become a major challenge for the aquaculture industry, resulting in significant economic losses. The use of costly and toxic antibiotics for treatment has a negative impact on the aquatic environment. Consequently, there has been a growing interest in probiotics as a non-antibiotic approach to manage disease outbreaks and improve fish performance. The use of the yeast *Saccharomyces cerevisiae* (SC) has shown remarkable benefits in aquaculture. In February 2025, a systematic search was conducted based on the Web of Science (WoS) database for the period 2015–2025 to identify relevant studies investigating the beneficial effects of SC in aquaculture. After searching on WoS, 466 documents were found and analyzed using R-bibliometric package for comprehensive analysis to identify research gap, trends, and distribution of global literature that focuses on SC in aquaculture. The most relevant and recent articles were reviewed, summarized and discussed. The yeast SC have shown a wide range of benefits, including improved growth performance, feed efficiency, enhanced diversity of the gut microbiome and immune response. The implementation of SC is becoming a recent trend and its efficacy in aquatic environments has been thoroughly investigated. This review aims to provide a valuable insight into SC as one of the most important aquaculture probiotics. It also emphasizes the need for further research to fully understand its benefits and the way it works.

## 1. Introduction

Aquaculture is one of the fast-growing animal food industries, supplying approximately 94 million tons of seafood and providing 15% of the world’s animal protein [1]. It plays a significant role in the global food supply [2,3]. Since 2001, the global aquaculture industry has shown a consistent annual growth rate of 5.8%, reflecting an increased demand for animal protein in rapidly growing economies. The aquaculture sector is largely driven by Asia, which accounts for 90% of global production. In 2016, China alone contributed more than 61% [4].

The occurrence of pathogens such as *Vibrio anguillarum*, *V*. *harveyi*, *Aeromonas hydrophila*, *A*. *salmonicida*, *Flavobacterium psychrophilum*, *Yersinia ruckeri*, *Pseudomonas fluorescens*, and *Citrobacter freundii* has been noted and could negatively impact the production of fish and other aquaculture species [5]. These pathogens and poor environmental factors are harmful to productivity and result in serious financial loss for aquaculture farmers. Fish practices, such as overfeeding, overcrowding, and water contamination, also contribute to the presence and spread of pathogens in aquatic environments [6,7,8].

Aquaculture frequently uses antibiotics for two primary reasons: to prevent and treat diseases, and to enhance fish performance [9,10]. However, the extensive use of antibiotics in fish farming has created conditions that favor the growth of drug-resistant bacterial strains, which can spread rapidly [11]. These resistant bacteria can negatively affect aquaculture production, fish consumers, and the surrounding environment [12,13,14]. Alternatively, probiotics have been utilized as an eco-friendly approach to enhance aquaculture sustainability [15,16,17].

The term “probiotic” is derived from the Greek words “pro” and “bios”, which together mean “for life” [18]. However, the initial definition of a probiotic was put forth by Parker [19], who defined a probiotic microorganism as one that contributes to intestinal microbial balance. According to the World Health Organization (WHO) and the Food and Agriculture Organization (FAO), probiotics are “live microorganisms which, when administered in adequate amounts, confer a health benefit to the host” [20]. A large number of studies have shown that probiotics are effective for a variety of fish and aquatic animals, such as African catfish (*Clarias gariepinus*), European sea bass (*Dicentrarchus labrax*), Nile tilapia (*Oreochromis niloticus*), Rainbow trout (*Oncorhynchus mykiss*), rohu (*Labeo rohita*), Indian Major Carp (*Labeo rohita*), snook (*Centropomus undecimalis*), common carp (*Cyprinus carpio*), and red seabream (*Pagrus major*) [21,22,23,24,25]. Among the probiotics, *Saccharomyces cerevisiae* is widely recognized as an important player in various domains of animal health, nutrition, and biotechnology.

The protein-rich probiotic *Saccharomyces cerevisiae* [26], due to its cellular components (e.g., β-glucan, glucooligosaccharides, mannooligosaccharides, and enzymes), has emerged as the most commonly used yeast in the aquaculture industry with multifaceted benefits for improving the health and productivity of various aquaculture species [27,28]. The probiotic potential of *S. cerevisiae* is supported by its resistance to various environmental stresses. Studies show that *S. cerevisiae* can survive in a variety of temperatures, making it a good choice for aquaculture, where temperatures can fluctuate [29]. Additionally, its tolerance of acidity and production of antimicrobial compounds provide an extra layer of protection against pathogens in aquatic environments. The secretion of antimicrobial peptides has been demonstrated in competitive interactions with non-*Saccharomyces* yeast strains, ensuring *S. cerevisiae*’s dominance during fermentation processes [30]. The various studies on the use of *S. cerevisiae* yeast in aquaculture have not been fully incorporated [31].

Therefore, the current study aims to discover the potential impact of *S. cerevisiae* in aquaculture based on the latest published scientific articles (2024–2025). It also aims to provide a bibliometric analysis of trends in the use of this yeast supplement in aquaculture. This analysis was designed to identify trends, limitations, and research gaps in the existing literature concerning the interaction between *S. cerevisiae* and other probiotics and prebiotics in aquaculture under various environmental conditions.

## 2. Results and Discussion

### 2.1. Situation of the Scientific Research on Saccharomyces cerevisiae (SC) in Aquaculture Based on WoS Database

#### 2.1.1. Growth of SC-Related Documents 2015–2025

**Figure 1:**
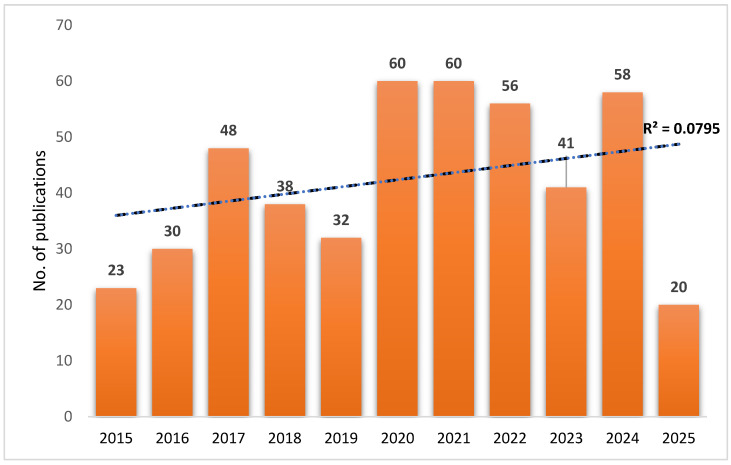
Growth of scientific publications of SC-related documents in aquaculture, based on the WoS database. The correlation coefficient (R^2^) of the exponential curve between 2000 and 2025 was 0.0795.

The number of published documents about the yeast probiotic SC in aquaculture covering the period between 2015 and 2025 was 466 in total. The publication trend demonstrates an increasing interest in the yeast SC, particularly after 2016. A notable increase occurred in 2020 and 2021, each recording 60 (12.88%) publications, suggesting a surge in research activity possibly driven by growing recognition of the potential use of SC in enhancing fish health and performance. In 2021, Dawood et al. [32] reported that *S. cerevisiae* in fish feeds aligns well with current trends in the aquaculture sector, emphasizing the shift away from antibiotics and towards more natural health-promoting alternatives. Although slight declines were observed in 2019 (32 publications, 6.87%) and 2023 (41 publications, 8.8%), interest increased quickly in 2024 with 58 publications (12.45%). The sharp drop to 20 (4.29%) publications in 2025 is likely due to incomplete data for the current year. Despite year-to-year variability, the fitted linear trend line (R^2^ = 0.0795) (Figure 1) indicates a modest but consistent growth in the number of publications over the past decade. This trend reflects a sustained and expanding research focus on SC as a functional probiotic and prebiotic in aquaculture, underscoring its growing relevance in fish nutrition, immunity, and disease resistance.

#### 2.1.2. Leading Countries on SC Research in Aquaculture

In total, 52 countries have published at least one SC-related document on the WoS. In fact, 466 documents have been published by these 52 countries. Table 1 presents the distribution of SC-related publications in aquaculture by country, highlighting both the volume and nature of international collaboration. China alone leads with 84 publications, accounting for 18.02% of the total output, followed by Egypt (10.30%) and Brazil (7.08%). These top-contributing countries indicate strong regional research activities, especially in Asia and South America. China also showed the highest number of single-country publications (SCP = 63), indicating a substantial volume of domestic research, while maintaining international collaboration through 21 multi-country publications (MCP), corresponding to 25% of its total. In fact, China is responsible for about 35% of the world’s fish and seafood production, making it the world’s largest producer of aquatic species [33]. China’s aquaculture industry is characterized by its vast size and diversity. In 2020, it produced over 49 million tons of fish. This includes not only finfish, but also mollusks and crustaceans, allowing for a wide range of products that meet the demands of both domestic and international markets [33].

In contrast, countries like Sweden (57.14%), Bangladesh (45.45%), Malaysia, and Norway (each 63.63%) exhibited relatively high MCP%, reflecting a strong inclination toward international collaboration despite their lower overall output. Japan stands out with a 100% MCP, suggesting that all its contributions are co-authored internationally. Similarly, countries like Australia (66.66%) and Pakistan (55.55%) showed higher proportions of collaborative research, underscoring the importance of global partnerships in advancing this niche field. Overall, the data indicate that while certain countries dominate in terms of quantity, others play significant collaborative roles in the global research network on Saccharomyces cerevisiae in aquaculture.

**Figure 2:**
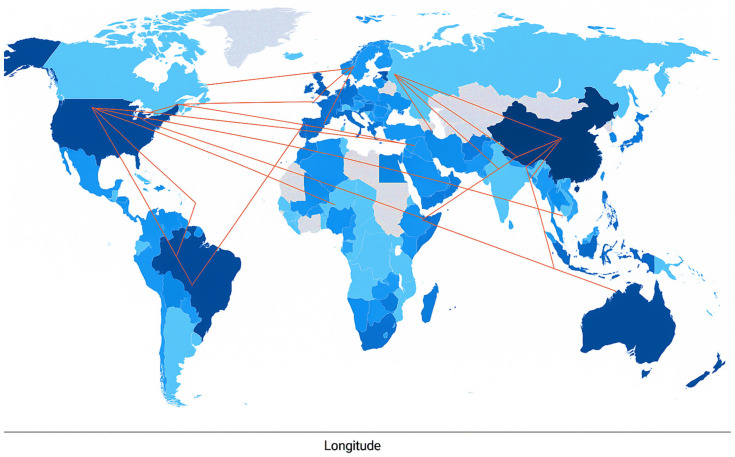
Patterns of international collaboration on publications related to *Saccharomyces cerevisiae* in aquaculture (2015–2025). Countries with darker colors indicate higher productivity. The linking lines represent collaboration between countries.

The international collaboration network among countries contributing to the SC research in aquaculture is presented in Figure 2. This map shows the global network of countries collaborating on SC-related aquaculture research. The intensity of the blue color represents the volume of publications, with darker colors indicating a higher research output. China, Egypt, Brazil, and the United States are notable contributors with relatively high publication counts. Red lines connecting countries indicate international co-authorships, reflecting research collaboration. The United States, China, and European nations are strongly interconnected, acting as major focal points in the global research network. Despite having fewer total publications, countries like Japan, Malaysia, and Norway demonstrate strong international engagement, as evidenced by their high proportion of multi-country publications (MCPs). This finding aligns with our previous bibliometric analysis, which showed that China, Egypt, and the USA are the most prominent countries in aquaculture probiotic research [15].

The map also reveals a geographically diverse and interconnected research landscape in which emerging economies, such as Egypt, India, and Brazil, collaborate with established research leaders. This trend emphasized the increasing global significance of probiotics in aquaculture and the collaborative nature of the current scientific investigation into SC as a functional feed supplement and health enhancer in aquatic animals.

#### 2.1.3. The Core Sources, and Most Cited Publications on SC Research

**Figure 3:**
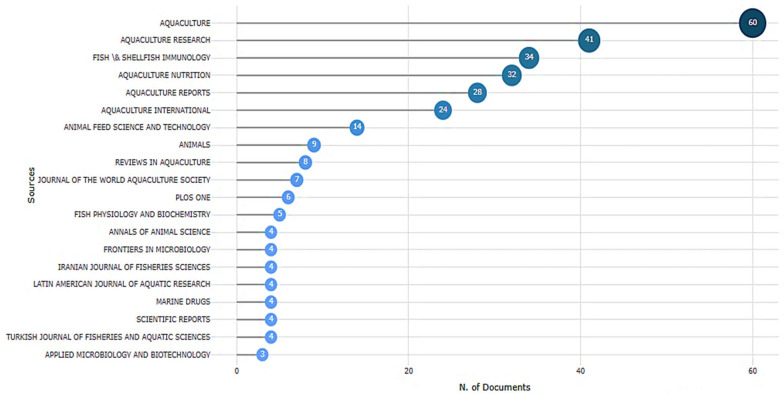
Top journals publishing research on SC-related documents (2015–2025) based on the WoS database. SC: *Saccharomyces cerevisiae*.

The use of *Saccharomyces cerevisiae* (SC), a beneficial yeast, has gained increasing focus in aquaculture due to its probiotic and synbiotic properties. Figure 3 shows the distribution of documents related to the application of SC in aquaculture across various scientific journals. *Aquaculture* was the most prominent source, with 60 publications, followed by *Aquaculture Research* (41 publications) and *Fish & Shellfish Immunology* (34 publications). These findings showed the important role these journals play in sharing aquaculture and fish health research. Additional journals, including *Aquaculture Nutrition*, *Aquaculture Reports*, and *Aquaculture International*, also made significant contributions, reflecting a rising interest in nutritional and health-related aquaculture topics. Specialized journals such as *Fish Physiology and Biochemistry* and *Frontiers in Microbiology* suggest that this research topic intersects with disciplines such as *microbiology* and *fish physiology*. The presence of multidisciplinary journals, such as *PLoS ONE* and *Scientific Reports*, has pointed to broader scientific interest and applicability. The dominance of aquaculture-specific journals emphasizes the maturity and specialization of the field, while contributions from related fields indicate the interdisciplinary nature of current research trends. The wide distribution of SC-related documents in various journals reflects the growing importance of this research topic. This aligns with the findings of Tucciarone et al. [34], who reported a rise in publications on sustainable aquaculture based on a recent trend analysis.

**Figure 4:**
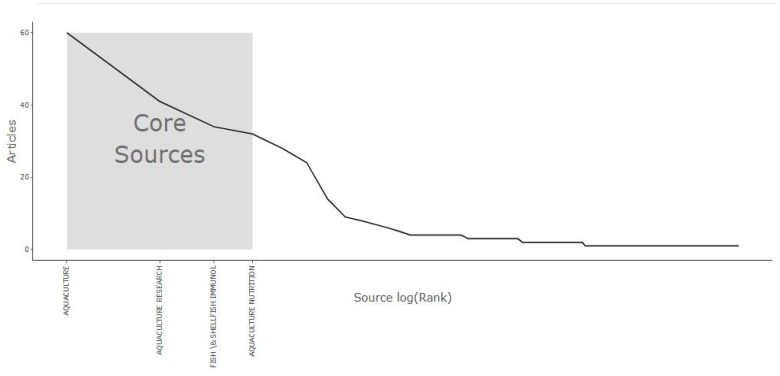
Bradford’s principle application: identifying core journals in SC-related documents in aquaculture. Based on the WoS database (2015–2025). SC: *Saccharomyces cerevisiae*.

Figure 4 introduces the core journals that published research on SC-related documents. The plot shows a decline in the number of articles beyond the first few journals, indicating a concentration of research output within a few sources, according to Bradford’s principle (a small number of sources account for most publications on a topic) [35]. Specifically, *Aquaculture*, *Aquaculture Research*, *Fish & Shellfish Immunology*, and *Aquaculture Nutrition* were identified as core sources within the shaded region of the graph. The sharp drop in productivity among the remaining journals supports Bradford’s principle. This finding aligns with the results shown in Figure 3, confirming the importance of these core journals in aquaculture and fish health research. Understanding the distribution of publications helps researchers prioritize target journals for their work and identify influential platforms that shape the academic landscape in this area.

Table 2 presents the 20 most-cited articles in the research domain, emphasizing influential studies that have significantly impacted the field. The two most-cited papers are by Dawood et al. (2016, *Aquaculture*) [36] and Oberbeckmann et al. (2016, *PLOS ONE*) [37]. Each paper has received 372 total citations, averaging 37.2 citations per year, which indicates their foundational role in the field. Hai (2015, *Journal of Applied Microbiology*) [38] and Carbone (2016, *Fish & Shellfish Immunology*) [39] achieved similar citation counts, reflecting sustained academic interest in their research on microbiota modulation and host immune responses.

Several papers published by Dawood appeared in the top 20 list, indicating his significant contributions to research on probiotics and functional feeds in aquaculture. Additionally, recent publications, such as Rohani M.F. (2022, *Fish* & *Shellfish Immunology*) [46], demonstrated strong annual citation rates (39.25 citations per year), indicating emerging research that is rapidly gaining recognition. The dominance of journals such as *Fish & Shellfish Immunology*, *Aquaculture*, and the *Journal* of *Applied Microbiology* among these highly cited works aligns with earlier identification of these sources as core journals (Figure 3 and Figure 4). This further supports their central role in disseminating impactful research. This citation analysis helps to identify key authors and studies that have laid the groundwork for future investigations into functional diets, host–microbe interactions, and immune modulation in aquaculture species.

### 2.2. The Potential Use of the Yeast SC in Aquaculture Based on Keyword Analysis

#### 2.2.1. Dendrogram of Keyword Clusters

**Figure 5:**
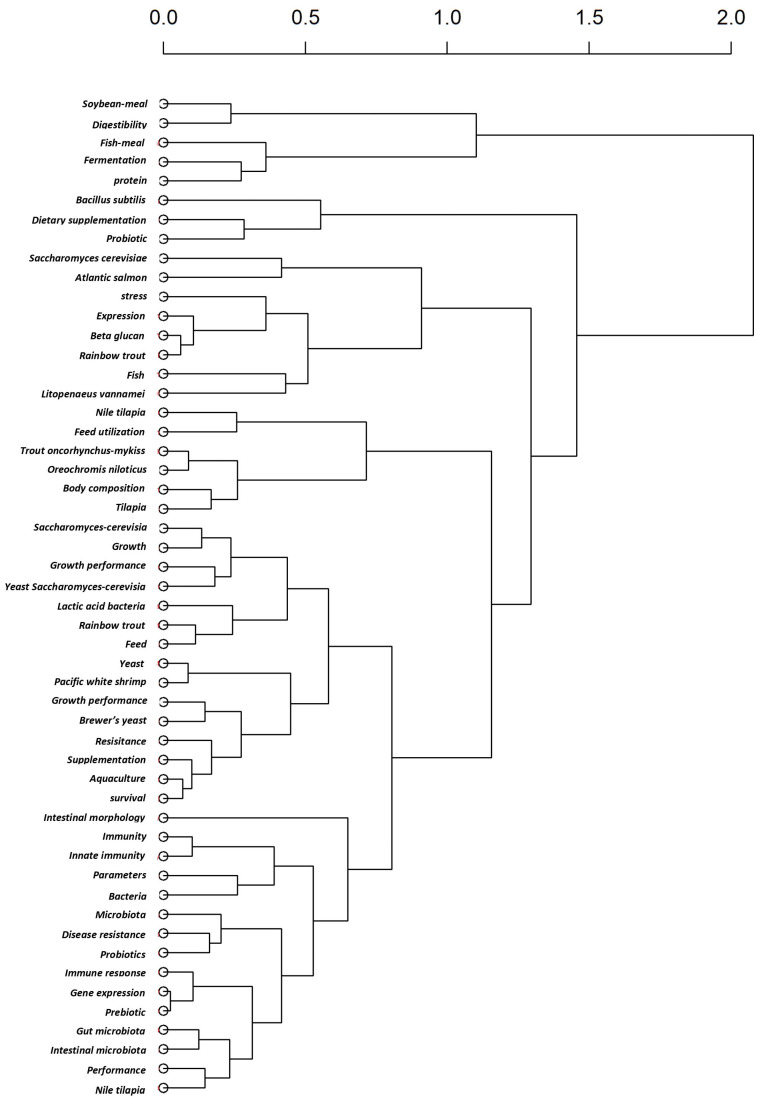
Dendrogram of keyword clusters based on factorial analysis of the yeast SC-related document in aquaculture (2015–2025) based on the WoS database.

The dendrogram, derived from a factorial analysis of author keywords (Figure 5), shows how key terms in the literature cluster together based on their co-occurrence. This hierarchical clustering approach reveals the thematic structure of the research domain. Keywords that are closely linked form clusters that represent distinct research subfields, and the branching height indicates the degree of dissimilarity between groups of keywords. The analysis identified multiple thematic clusters. For example, terms such as probiotics, gut microbiota, and immune response frequently appear together, indicating a significant research focus on the health advantages of the yeast SC in aquaculture species.

The addition of yeast-based probiotics resulted in notable improvements in growth metrics and strengthened immune responses against pathogens, including *Aeromonas* spp. [27], *Vibrio* spp. [56], and *Citrobacter* spp. [57]. Other clusters may reflect related themes, such as growth performance, digestive enzyme activity, histomorphology, and disease resistance. These clusters indicate that the literature is organized around several interconnected yet distinct research trends.

#### 2.2.2. Thematic Map Analysis

**Figure 6:**
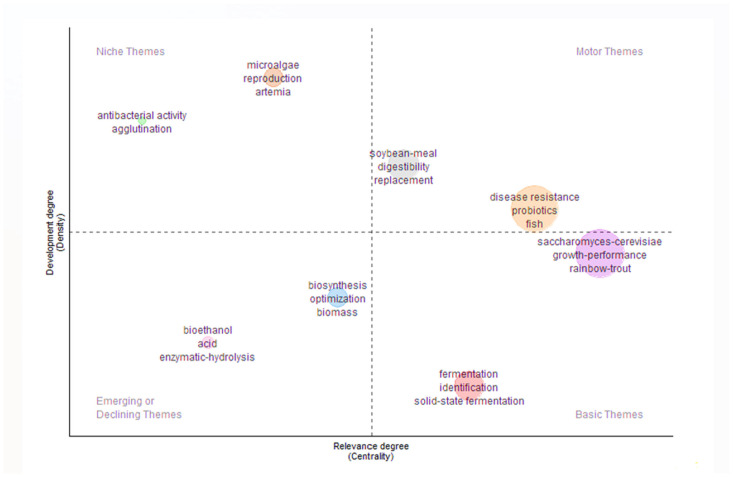
Thematic map of aquaculture probiotic research (1999–2020) based on Web of Science data. Bubble size represents keyword frequency, and position is based on centrality and density.

The density of the two largest thematic clusters, “disease resistance, probiotics, and fish” and “*Saccharomyces cerevisiae*, growth performance, and rainbow trout”, revealed two distinct interconnected areas of research within aquaculture studies (Figure 6). The first cluster emphasizes the role of probiotics in enhancing disease resistance across various fish species, reflecting a broad and foundational theme and indicating a strong interest in health management and sustainable aquaculture practices. Its high density suggests a substantial body of research and collaboration on probiotic use and immune response in general. The second cluster, centered on *S. cerevisiae* and its impact on the growth performance of rainbow trout, points to a more specific line of inquiry. Its density highlights concentrated research efforts on this particular yeast probiotic. Numerous studies have investigated the effects of yeast on rainbow trout (*O. mykiss*). These studies have found that the dietary inclusion of *S. cerevisiae* improves intestinal microbiota composition and growth performance [28,58]. The coexistence of these dense themes underscores a layered research landscape in which general health-promoting strategies and species-specific probiotic applications are both critical to advancing aquaculture science.

#### 2.2.3. Insights from the Three-Field Plot

**Figure 7:**
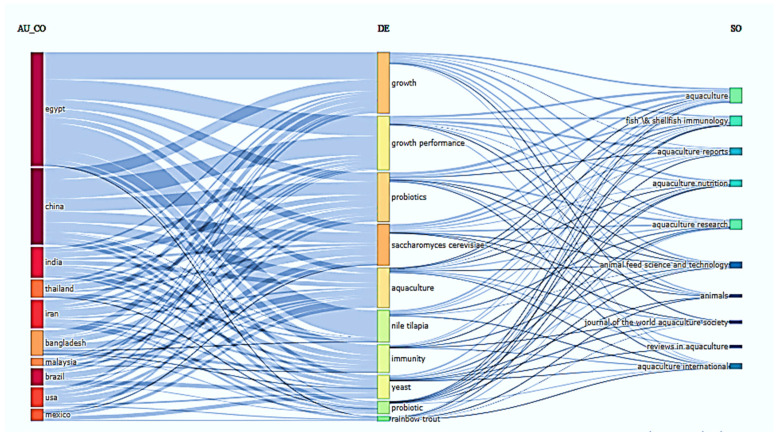
Three-field plot illustrating the relationships between contributing countries (AU_CO), author keywords (DE), and journals (SO) in research on the use of *Saccharomyces cerevisiae* and probiotics in aquaculture.

The three-field plot highlights the growing global interest in the application of the probiotic *S. cerevisiae* within aquaculture, based on keyword co-occurrence across countries, research themes, and journals (Figure 7). Notably, Egypt, China, and India emerge as leading contributors to this area, reflecting a strong regional research focus. The central linkage of *S. cerevisiae* with key terms such as “growth”, “growth performance”, “probiotics”, and “immunity” underscores its potential as a multifunctional additive in fish diets. Moreover, its association with economically important species like Nile tilapia and rainbow trout, and frequent publication in prominent journals such as *Aquaculture*, *Aquaculture Nutrition*, and *Fish & Shellfish Immunology*, further emphasizes its significance in advancing both the nutritional and immunological dimensions of aquaculture. Collectively, the three-field plot illustrates how *S. cerevisiae* is becoming an integral component of sustainable and health-oriented aquafeed strategies.

#### 2.2.4. Insights from the Trend Topics

**Figure 8:**
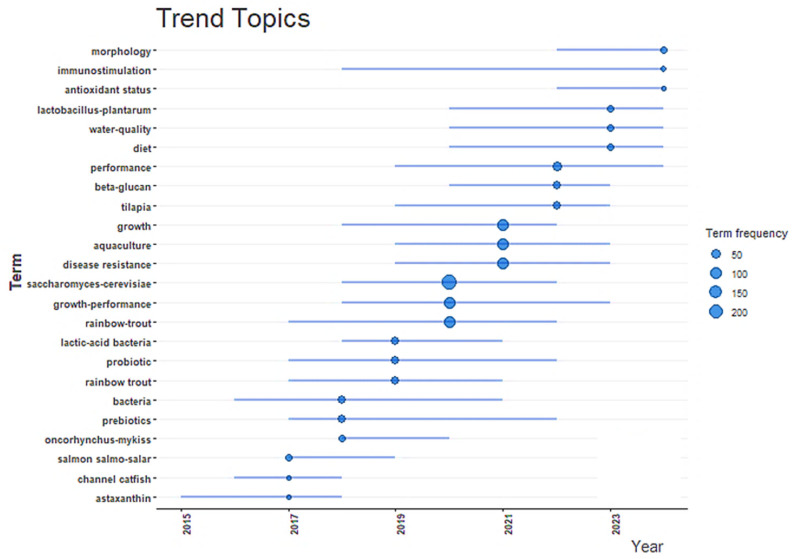
Trend topic analysis showing the temporal evolution and frequency of key terms related to *Saccharomyces cerevisiae* in aquaculture (2015–2025).

Trend topic analysis reveals an increasing research interest regarding *S. cerevisiae* and its role in aquaculture, especially since 2019 (Figure 8). The appearance of this term alongside high-frequency keywords such as “growth”, “disease resistance”, “probiotic”, and “growth performance” indicates a clear thematic convergence on using this yeast to enhance the health and productivity of aquatic species. Associated topics such as “antioxidant status”, “immunostimulation”, and “beta-glucan” suggest an expanding scope of investigation into the functional and immunomodulatory properties of *S. cerevisiae*. The alignment of these terms with economically significant species, such as tilapia and rainbow trout, reflects their practical relevance in industry-driven research. This temporal trend indicates the emergence of *S. cerevisiae* as a key component in sustainable aquaculture strategies, particularly in the context of antibiotic alternatives and functional feed innovations. Its use contributes to healthier aquaculture systems and aligns with current global objectives aimed at reducing environmental impact of antibiotics and promoting food safety and aquaculture sustainability [31].

### 2.3. Use of Saccharomyces cerevisiae (SC) in Aquaculture Based on the Recent Published Documents (2024–2025)

#### 2.3.1. Enhancement of Growth, Feed Efficiency, and Digestibility

The main parameters used to evaluate the growth of animals after treatment with probiotic-based feed include final body weight (FBW), feed conversion ratio (FCR), survival (%), and specific growth rate (SGR). The FCR value is calculated using the formula: FCR = Feed intake/(Wf − Wi), where Wf is the final body weight (g), and Wi is the initial wet body weight (g). SGR is determined by the formula: SGR = (lnWf − lnWi)/t × 100, where t is the time (days). The enhanced growth indices result in faster harvesting times and improved production efficiency, both of which are vital for meeting the increasing global demand for seafood [15,59].

Numerous studies discussed the beneficial impact of probiotic yeast *Saccharomyces cerevisiae* (SC) on growth performance of different aquatic animals. For instance, rainbow trout (*O. mykiss*) fed on supplemented diets (1 × 10^8^ CFU/g of SC) and 250–500 mg/kg for 4 weeks could enhance (*p* < 0.05) fish body weight and feed efficiency [60]. A report from China stated that the growth performance (weight gain, FCR, and body weight) of channel catfish (*Ictalurus punctatus*) can be significantly (*p* < 0.05) improved when animals received diet supplemented with the yeast *S. cerevisiae* for 12 weeks [27]. In addition, the FCR value and growth rate of Striped Catfish (*Pangasianodon hypophthalmus*) can be significantly improved when fish receive a diet supplemented with freeze-dried microencapsulated SC for 120 days [61]. Similarly, a report from Indonesia indicated that the supplementation by *S. cerevisiae* at 10 g/kg could significantly (*p* < 0.05) increase protein efficiency, and the growth rate of Saline Red Tilapia (*Oreochromis* spp.) when fish (1.07 ± 0.07 g) received a diet supplemented with yeast probiotic for 28 days [62]. Recently, extensive research studies have been performed on a wide range of aquaculture species, especially fish and shrimp, to evaluate the impact of probiotic yeast *S. cerevisiae* on growth parameters and feed efficiency (Table 3).

Many studies have confirmed a strong correlation between probiotics and fish growth parameters. However, the specific mechanisms by which probiotics improve growth parameters are not fully understood. As reported by Eliopoulos et al. [63] and Yang et al. [64], probiotics are important for increasing feed palatability via fermentation. This improvement can be linked to their ability to modify the sensory properties of feed. For example, certain probiotic strains ferment feed and produce metabolites that enhance its aroma, which plays an essential role in sensory perception, making feed more appealing to animals. Yang et al. [64] showed that probiotic fermentation can effectively enhance the palatability of traditional Chinese herbs by improving their flavor and taste. Furthermore, a recent study showed that the flavor and palatability of plants can be altered by fermentation and subsequently stimulate animals’ appetites through the formation of aroma and flavor compounds [65].

#### 2.3.2. Impact on Disease Resistance, Immune Response, and Gut Integrity

In addition to enhancing growth parameters, the integration of probiotic *S. cerevisiae* in aquaculture has gained considerable attention for its ability to enhance disease resistance, modulate the immune system, improve gut integrity, and enhance microbial diversity within the gut of diverse fish and shrimp species (Table 3). Therefore, it fosters overall health in aquatic animals and contributes to the sustainability of aquaculture.

In the context of disease resistance and immune response, Mohammed et al. [66] examined the effect of *S. cerevisiae* fermented product (Diamond V Original XPC) on hybrid catfish (*Ictalurus furcatus* × *I. punctatus*). They noticed that marginally increased resistance to columnaris disease, enhanced level of immune effectors in the serum, such as lysozyme, complement, and immunoglobulin, were found when fish received a yeast-supplemented diet for 6 weeks. In addition, crayfish (*Procambarus clarkia*) exhibited significantly (*p* < 0.05) upregulated levels for prophenoloxidase and lysozyme when fish received a diet supplemented with *S. cerevisiae* YFI-SC2 at 10^7^ CFU/g for 28 days. Additionally, the resistance against *Citrobacter freundii* can be enhanced by *S. cerevisiae* at 10^7^ CFU/g feed supplementation. Furthermore, supplementation with *S. cerevisiae* could reduce the expression of intestinal inflammatory factors, and remarkably improve resistance to *Vibrio harveyi* infection in orange-spotted groupers (*Epinephelus coioides*) [67], resistance to *A. hydrophila* of ornamental fish (*Poecilia latipinna*) [68], resistance to a mixture of *A. hydrophila* NJ-1 and *A. veronii* HM091 of channel catfish (*Ictalurus punctatus*) [27], resistance to *A. hydrophila* AH2 in Indian major carp (*Labeo rohita*) [69], resistance to *Streptococcus agalactiae* in tambaqui (*Colossoma macropomum*) [70], and resistance of Nile tilapia (*O. niloticus*) to the sudden exposure to high water temperature (40 °C) [71].

Regarding gut health and microbiota, numerous research works have shown the potential use of *S. cerevisiae* for improving intestinal microbiota and gut health (Table 3). For example, El-Mokhlesany et al. [72] noticed that when mullet (*Liza ramada*) received diet contaminated with mycotoxins (AFB_1_) at 1 mg/kg and were supplemented with *S. cerevisiae* (5 × 10^6^ cells/g), a remarkable improvement was recorded with regard to their intestinal structure and gut health in comparison to those fed solely an AFB_1_-contaminated diet. In addition, the length, width, and villus area of Nile tilapia (*O. niloticus*) can be significantly increased when fish receive a diet supplemented with *S. cerevisiae* at 4 g kg^−1^. Likewise, intestinal mucosal fold, width of lamina propria, width of enterocytes, and number of goblet cells can be increased [73]. Similarly, immunity and gut microbiota of Nile tilapia (*O. niloticus*) reared in low-input ponds can be altered under *S. cerevisiae* feed supplementation after 180 days [74]. Furthermore, the administration of the microencapsulated probiotic yeast *S. cerevisiae* has been correlated with an enhancement of length and width of the intestinal folds, and intestinal microbiota [75]. Taking all this into account, the improvement of the histomorphological indices of intestinal villi is directly correlated to the absorption and digestion efficiency [76]. However, the specific mechanisms by which probiotics and prebiotics affect intestinal absorption are not clear. The cells at the end of the villi are constantly sloughed off, and the intestinal epithelium’s renewal rate is high enough to replace them [77]. Moreover, the beneficial effect of *S. cerevisiae* on intestinal histomorphology could be attributed to the monosaccharides that compose its cell wall.

#### 2.3.3. The Synbiotic Effect

Integrating the yeast probiotic *S. cerevisiae* into a synbiotic strategy gives aquaculture practitioners a powerful means of improving health and productivity of various fish species under various aquaculture systems (Table 3). For example, Siddik et al. [78] reported that the growth indices, immune response (TNF-alpha and IL-10), intestinal goblet cells, microvillous length, and gut health of barramundi (*Lates calcarifer*) juveniles remarkably improved when fish received co-supplementation of *S. cerevisiae* and *Lactobacillus casei*. Additionally, Vidakovic et al. [79] noticed that the combination of yeasts (*Wickerhamomyces anomalus* and *Saccharomyces cerevisiae*) can replace 40% of fishmeal for rainbow trout (*Oncorhynchus mykiss*) without affecting growth performance, intestinal health, or nutrient digestibility.

Furthermore, a combined probiotic containing *Bacillus subtilis* (1.5 × 10^9^ CFU/g), *Aspergillus oryzae* (2 × 10^9^ CFU/g), and *S. cerevisiae* (10^9^ CFU/g) has beneficial effects on Nile tilapia (*O. niloticus*) juveniles. These probiotics provide resistance against *Aeromonas hydrophila* and *Streptococcus iniae* after three weeks of challenge, growth rates, and feed conversion improvements [48].

**Table 3:** The significant role of the yeast *Saccharomyces cerevisiae* in aquaculture based on the most recent published research papers (2024–2025) on WoS.

Country	Host	Experimental Conditions	Key Findings	Date	Citation
Pakistan	Pacific whit shrimp (*Litopenaeus vannamei*)	▪ 42-day trial ▪ IBW: 108 ± 0.19 g ▪ Feeding rate: 5% of biomass	▪ SGR: Prob. ↑ CD: ↓ ▪ PE ratio: Prob. ↑ CD: ↓ ▪ Red blood cell: Prob. ↑ CD: ↓ ▪ Survival: Prob. ↑ CD: ↓ ▪ Serum lysozyme: Prob. ↑ CD: ↓ ▪ Leukocytes: Prob. ↑ CD: ↓	2025	[80]
Canada	Atlantic Salmon (*Salmo salar* L.)	▪ 5–6-week trial ▪ Challenging with *Tenacibaculum maritimum* and *Moritella viscosa*	▪ Mortality reduced by *S. cerevisiae* yeast β-glucan (0.1%) after challenging with *T. maritimum* and *M. viscosa*	2025	[81]
Portugal	Green macroalga (*Ulva rigida*)	▪ Fermentation by *S. cerevisiae* was performed ▪ A functional analysis was conducted	▪ Antioxidant properties ↑ ▪ Protein bio-accessibility ↑	2025	[82]
UK	Atlantic Salmon (*Salmo salar* L.)	▪ 8-week trial ▪ Yeast-derived functional feed additives were tested to mitigate the effects of soybean meal-induced enteritis (SBMIE)	▪ Severity of SBMIE ↓ ▪ Intestinal morphology ↑ ▪ Goblet cell hyperplasia ↓ ▪ Expression of *casp3b* and *pcna* ↓ ▪ Gut health ↑	2025	[83]
Thailand	Nile tilapia *(Oreochromis* *niloticus*)	▪ 56-day trial ▪ IBW: 5.22 ± 0.02 g ▪ 4 diets containing different levels of fermented rice bran (FRB) at 0, 100, 200, and 300 g/kg	▪ In FRB groups, amylase and protease ↑ ▪ In all FRB groups, survival (%) after challenging with *Streptococcus agalactiae* for 10 days: ↑ ▪ In FRB20% and FRB30%, expression of insulin-like growth factor transcripts, non-specific immunity ↑ ▪ In FRB30% villus height ↑	2025	[84]
Australia	Barramundi (*Lates calcarifer*)	▪ 56-day trial ▪ IBW: 4.25 ± 0.09 g ▪ Two seaweed-based diets, probiotic fortified *Sargassum linearifolium* (PF-SL) and *S. linearifolium* without probiotic fortification (SL), were compared with a standard barramundi feed (control)	▪ In PF-SL group: mucus cell ↑, size of mucus cells ↑, skin mucosal barrier ↑, microvilli length ↑, fold length ↑, density of mucous cells ↑, submucosa width NE ▪ Also, the immune cells such as rodlet cells (number/fold) and intraepithelial lymphocytes (number/fold) were ↑ in PF-SL group	2025	[85]
Iran	Rainbow trout (*Oncorhynchus mykiss*)	▪ 60-day trial ▪ IBW: 31.6 ± 0.33 g ▪ diet with 15% dietary cotton seed meal (CSM) + 1 × 10^8^ CFU/g of *S. cerevisiae* (CSMY) ▪ diet with 15% CSM plus 1 × 10^8^ CFU/g of iron-enriched *S. cerevisiae* (CSMYFE)	▪ Growth performance: NE ▪ FCR ↓ ▪ Leukocyte counts: NE plasma iron concentration, alanine, aminotransferase, and hepatic antioxidant: NE ▪ histological parameters: NE ▪ CSMYFE, CSMY, and the control are same	2025	[86]
Canada	Zebrafish (*Danio rerio*)	▪ 63-day trial ▪ Age: 3–4 months ▪ The study investigated the effects of yeast probiotics, prebiotics, a postbiotic (butyrate), and black soldier fly larvae (BSFL) meal	▪ weight gains: NE ▪ Expression of *angiopoietin-like 4* in yeast probiotic ↓ ▪ In all groups, *TNF-α, IL-1β, hepcidin, NF-κB/p65 and NF-κB/p65* ↑	2024	[87]
Italy	Gilthead Seabream (*Sparus aurata*)	▪ Over 135-day trial ▪ 60% replacement of fish meal by a blend of plant, yeast (*S. cerevisiae*) and krill meal feed ingredients	▪ Growth performance in *S. cerevisiae* supplemented diet ↑ ▪ Swimming activity in *S. cerevisiae* group ↑	2024	[88]
Egypt	Nile tilapia *(Oreochromis niloticus*)	▪ 90-day trial ▪ The study assessed the effects of diets containing plant protein (cottonseed meal, sunflower meal, and jojoba meal) fermented with *S. cerevisiae* (SC) at three concentrations (25%, 50%, and 75%) instead of fishmeal (FM)	▪ SC at 25% and 50%, and the control: body weight ↑, weight gain ↑, SGR ↑, and daily gain ↑, whereas fish fed 75% these parameters were ↓ ▪ Also, the control diet, SC 25%, and SC-50%: complement component (C3) ↑, C4 ↑, growth hormone ↑, and IgM ↑ ▪ FM can be replaced with either SC−25% and SC−50% without affecting feed utilization and growth parameters	2024	[89]
Egypt	Nile tilapia *(Oreochromis niloticus*)	▪ 70-day trial ▪ IBW: 5.19 ± 0.02 g ▪ The study uses *Lactobacillus rhamnosus* and *S. cerevisiae*) to ferment sugar beet bagasse waste then used as fish feed	▪ Nitrite and ammonia ↓ ▪ Zooplankton community ↑ ▪ SGR ↑ ▪ Globulin, albumin, total protein, and IgM ↑	2024	[90]
Egypt	common carp (*Cyprinus carpio*)	▪ The study tested the effects of dietary *Chlorella vulgaris* (CV) and *S. cerevisiae* (SC) ▪ Fish received free CV and SC diet, CV-supplemented diet (5%), SC-supplemented diet (0.05%; (10 × 10^9^ CFU/g), or a mixture of both (5% CV + 0.05% SC)	▪ In CV or SC, growth performance ↑, protein efficiency ratio ↑, FCR ↓, serum total protein ↑, triglyceride ↓ ▪ SC, CV and SC mixture villi length ↑	2024	[91]
Peru	freshwater prawn (*Cryphiops caementarius*)	▪ The study investigated the effect of the activated yeast (*S. cerevisiae*) (SC), crude chitin (CC), and chitosan (CS)	▪ In SC group, growth performance ↑, total hemocytes ↑, semigranular ↑, and granular ↑ ▪ In SC group also, acid phosphates activity ↑, glutathione-S-transferase ↑, hemolymph clotting time ↓	2024	[92]
Iran	Zebrafish (*Danio rerio*)	▪ 60-day trial ▪ IBW: 80 ± 1.0 mg ▪ The study examined the impact of two fungal probiotics, *S. cerevisiae* 10^7^ CFU (SC) and *Aspergillus niger* (AN) with extracts of Jerusalem artichoke and white button mushroom	▪ In all groups except the control, SGR ↑, final weight ↑, protein efficiency ratio ↑, weight gain ↑, FCR ↓, protease ↑ ▪ In all treatment groups also, immune response was ↑	2024	[93]
Iran	Nile tilapia (*Oreochromis niloticus*)	▪ 8-week day trial ▪ IBW: 9.8 ± 0.1 g ▪ Four diets containing1 g/kg yeast (SC), 0.1 g/kg costmary essential oil (TB), a mixture of both (SC + TB), and without SC and TB (control)	▪ In SC, TB, and SC + TB groups, body weight ↑, SGR ↑, FCR ↓, and weight gain ↑ ▪ In all experimental groups also, amylase ↑, lipase ↑, protease ↑. But the highest SC + TB ▪ In addition, after challenges with *A. hydrophila* the survival rate in all exp. groups ↑ ▪ Furthermore, in SC + TB group: albumin ↑, globulin ↑, lysozyme ↑, cortisol ↓, glucose ↓, malondialdehyde ↓, and catalase ↓, before and after the challenge	2024	[94]
China	*Salmo trutta*	▪ IBW: 84.57 ± 1.83 g ▪ Three different probiotics have been used, *Saccharomyces cerevisiae* (SC), *Bacillus licheniformis* (BL), and *lactic acid bacteria* (LAB), and compared with the control diet (CON) ▪ Feeding: 3% of their body weight, twice daily	▪ Weight gain in SC and other probiotics groups was ↑ ▪ In all probiotics groups, the abundance of *Pseudomonas*, *Acinetobacter*, and *Rhizophagus* bacterial genera was similar to that in the top three comparative controls	2024	[95]
Brazil	Shrimp (*Penaeus vannamei*)	▪ 40-day trial ▪ The experiment had 5 treatments: clear water (CW) (control), *Bacillus* (B), *Bacillus* + *Lactobacillus* + *Pediococcus* (BLP), *Bacillus* + *Lactobacillus* + *Pediococcus* + Yeasts (*S. cerevisiae*) (BLPY), and *Bacillus* + Yeasts (BY)	▪ In BLP and CW groups: final weigh was ↑ than in the BY ▪ In BY group: abundance of nitrite-oxidizing bacteria was ↑ than BLPY, CW, and BY	2024	[96]
Malaysia	freshwater prawn (*Macrobrachium rosenbergii*)	▪ 3-week trial ▪ the study explored the effects of β-1,3/1,6-glucan derived from *S. cerevisiae* cell walls on the freshwater prawn post larvae (PL)	▪ At 0.2% β-glucan: wet weight ↑, survival ↑, total length ↑	2024	[97]
Egypt	Nile tilapia (*Oreochromis niloticus*)	▪ 8-week trial ▪ IBW: 4.24 ± 0.01 g ▪ The fish were fed diets supplemented with *S. cerevisiae* extract (SCE) (Hilyses) at concentrations of 0.0, 1.0, 2.0, or 3.0 g/kg	▪ SCE at 2 g/kg: final body weight ↑, weight gain ↑, SGR ↑, feed efficiency ratio ↑, protein efficiency ratio ↑, and energy utilization ↑ ▪ Hilyses supplementation: red blood cell count ↑, total leukocyte count ↑, hemoglobin concentration ↑, hematocrit ↑, and mean corpuscular volume ↑	2024	[98]
Egypt	*Mugil capito*	▪ 60-day trial ▪ IBW: 10.30 ± 0.10 g ▪ 4 groups were designed as follows: (1) a group fed a free probiotics (control), (2) a group containing *S. cerevisiae* (4 g/kg diet), (3) *L. bulgaricus* (2 g/kg diet), and (4) a diet containing a combination of both probiotics	▪ In group (4): superoxide dismutase ↑, catalase ↑, and glutathione peroxidase enzyme ↑ ▪ In all probiotic groups: glycogen storage ↑, melanomacrophage centers ↑, length of intestinal villi ↑	2024	[99]
Egypt	Nile tilapia (*Oreochromis niloticus*)	▪ 60-day trial ▪ IBW: 9.43 ± 0.08 g ▪ four treatments defined as control (no supplement) (T1), yeast *S. cerevisiae* (4 g/kg) (T2), garlic (30 g/kg) (T3), and a combination of both (T4)	▪ In T4: body weights ↑, weight gains ↑, and SGR ↑, defense against oxidative stress ↑ ▪ In T3 and T4: liver and intestinal histology ↑	2024	[100]
USA	white sturgeon (*Acipenser* *transmontanus*)	▪ The study investigated the effect of *Veronaea botryosa* (conidia), *V. botryosa* (mold), and a *S. cerevisiae* (yeast) immune responses of white sturgeon ▪ Animals injected with a virulent *V. botryosa*	▪ In mold or yeast groups: pro-inflammatory response upon challenge with virulent *V. botryosa* was ↑ compared to non-immunized fish ▪ In control group (animals not immunized but challenged with *V. botryosa*), the mortality was ↑	2024	[101]

“↑”: increase or upregulation at *p* < 0.05, “↓”: decrease or downregulation at *p* < 0.05, “IBW”: initial body weight, “CD”: control diet. “NE”: No effect.

### 2.4. Use of Machine Learning and AI

Integrating Artificial Intelligence (AI) and machine learning (ML) into the study of gut microbiota, with a particular focus on *S. cerevisiae* in aquaculture, holds significant potential for innovation. AI and ML techniques can improve our understanding of microbial interactions and functional dynamics within aquatic ecosystems. A key use of ML in aquaculture is predicting microbial community compositions and their functional interactions. In 2022, Nakanishi et al. [102] created a machine learning prediction model that used data from absorbance spectroscopy to estimate the density of cells in microbial mixtures, including *S. cerevisiae*. This predictive model is essential for optimizing conditions in aquaculture environments, where maintaining microbial balance is crucial for host health and optimal productivity.

Machine learning also provides innovative solutions for monitoring the health and efficiency of microbial activity in aquaculture. Machine learning-based profiling techniques can analyze gut microbiota and identify key microbial species, evaluating their functional roles. This assists in developing interventions that utilize *Saccharomyces cerevisiae* or similar microbes to improve the health of aquatic species [103]. Moreover, Palomba et al. [104] presented that the relevance of real-time monitoring in the context of bioprocesses was also underscored, with an emphasis placed on the importance of integrated systems using machine learning (ML) for the effective management of microbial populations.

On the whole, developing predictive models that use AI and ML is a reliable way to improve our understanding of gut microbiota behavior, especially with regard to probiotic applications such as *S. cerevisiae*. Combining functional genomics, microbiome analysis, and machine learning paves the way for significant improvements in aquaculture management practices, which aim to enhance the health and productivity of aquatic species.

## 3. Materials and Methods

### 3.1. Research Questions

**Figure 9:**
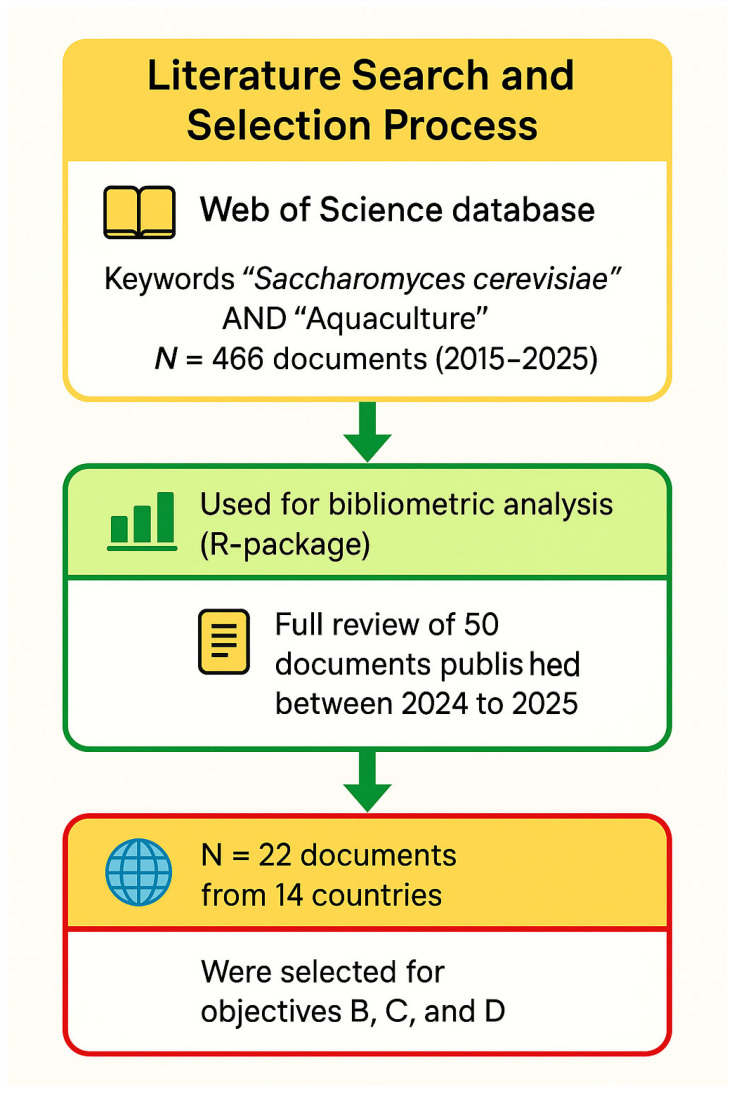
Workflow of document extraction, screening and processing using metadata obtained from the Web of Science database (WoS), 2015–2025.

- What are the main research areas, research quantity, global distribution of publications, and leading sources of studies focusing on the impact of the yeast *Saccharomyces cerevisiae* in aquaculture? This investigation was based on a selection of keywords, including “*Saccharomyces cerevisiae*” and “probiotics” (Figure 9).
- What are the effects of the yeast *S. cerevisiae* on the feed utilization and growth parameters of aquaculture species?
- What impact does *S. cerevisiae* have on disease prevention, survival (%), gut microbiome, and water quality in aquaculture species?
- What is the interactive effect between *S. cerevisiae* and other probiotics and prebiotics in aquaculture systems?

### 3.2. Searching Strategy

A comprehensive investigation was performed by examining the global literature in the Web of Science (WoS) database. WoS is considered the most comprehensive and well-known database for reviews and bibliometric analyses [15], which is why we selected it. The search was performed using a couple of keywords, including “*Saccharomyces cerevisiae*” and “Aquaculture” during the past decade (2015 to 2025). All documents written in English were considered. In addition, all research articles, review papers, or book chapters were also considered and counted; however, other types of documents were excluded. The search yielded a considerable amount of documents, which were 466 in total.

### 3.3. Data Processing and Bibliometric Analysis

For objective A, we exported the bibliometric parameters including authors, document titles, countries where the study was performed, publication dates, and author keywords, as BibTeX format from the WoS database. We performed in-depth analyses, including co-occurrence keyword assessment, citation analysis, trend, and factorial analysis, using RStudio v. 2025.05.0 + 496 (Boston, MA, USA) in connection with the bibliometric *R* package. The flexibility and statistical capabilities of R Studio v. 2025.05.0 + 496 (Boston, MA, USA) is essential for effectively visualizing complex and large amounts of data [105]. In brief, the metadata obtained from WoS database were converted to *BibTeX* format and uploaded to the R-bibliometric package for comprehensive analysis. The analysis yielded multiple knowledge maps illustrating the nature of emerging research of the yeast *S. cerevisiae* in aquaculture. The results and summary statistics were then exported to Microsoft^®^ Excel (Redmond, WA, USA) for organization and processing.

### 3.4. Narrative Review (2024–2025)

Regarding objectives B–D, data including country where study was conducted, animal host, growth indices, feed utilization efficiency, disease incidence, gut health and integrity, intestinal microbiome of animal host, immune parameters, level of ammonia and toxic nitrogen in water, and activity of digestive enzymes were extracted directly from the results of narratively reviewed articles or by using Web Plot Digitizer (Version 5.0) if the data were presented as figures. The results that were extracted were then presented in tables, and they were summarized and discussed.

## 4. Conclusions

The current review highlights the growing interest in the yeast *Saccharomyces cerevisiae* as a probiotic and prebiotic in aquaculture based on bibliometric analysis and applied scientific perspectives. The growing number of research publications from 2015 to 2025 reflects the global interest in sustainable, antibiotic-free methods for promoting the health and nutrition of aquatic animals. The bibliometric analysis of 466 documents using RStudio v. 2025.05.0 + 496 reveals strong international collaboration, particularly among China, Egypt, and Brazil. It also identifies core journals such as *Aquaculture* (60 publications), *Aquaculture Research* (41 publications), and *Fish & Shellfish Immunology* (34 publications), in addition to the influential publications that shape the current literature on yeast-based prebiotics and probiotics, such as papers by Dawood et al. (2016, *Aquaculture*) [36] and Oberbeckmann et al. (2016, *PLOS ONE*) [37]. The author’s keyword analysis identified multiple thematic clusters. For example, terms such as gut microbiota and immune response frequently appear together, indicating a significant research focus on the health advantages of the *S. cerevisiae* in aquatic species. The trend topic analysis showed an increasing interest regarding *S. cerevisiae* and its role in aquaculture, especially from 2019 to 2025. The appearance of high-frequency terms such as “growth”, “disease resistance”, and “growth performance” indicates a considerable interest in using this yeast to enhance the health and productivity of fish and other aquatic animals.

A recent narrative review confirms that *S. cerevisiae* supplementation contributes to enhanced growth performance, improved feed efficiency, stronger immune responses, better gut integrity, and higher disease resistance in various aquaculture species based on different case studies from different countries. Furthermore, its synergistic effects in synbiotic formulations highlight its potential as a multifunctional supplement in functional animal feed. Despite these advances, gaps remain in our understanding of the precise mechanisms through which *S. cerevisiae* exhibits probiotic effects, especially under different environmental conditions and when combined with other microbes. Emerging areas like AI and machine learning-based microbiome profiling are not well-represented in the current literature. These findings offer valuable insight on research trends and gaps and provide a foundation for future studies on the multifaceted role of SC in sustainable aquaculture.

Future research should clarify these mechanisms, optimize dosage and delivery methods, and evaluate long-term effects in commercial aquaculture systems. Continued integration of bibliometric tools and experimental evidence is essential to identify research gaps, guide research priorities, and maximize the benefits of *S. cerevisiae* in sustainable aquaculture. Future research directions should also focus on uses of multi-omics technologies, strain-specific functional analysis, and the integration of machine learning-driven approaches to better understand host–microbe interactions and optimize *S cerevisiae*-based formulations.

## Figures and Tables

**Table 1:** The top 20 producing countries of SC-related documents in aquaculture (2015–2025) based on WoS database. SC: Saccharomyces cerevisiae.

Country	No. of Articles	Articles %	SCP	MCP	MCP %
China	84	18.02	63	21	25.00
Egypt	48	10.30	27	21	43.75
Brazil	33	7.08	22	11	33.33
India	32	6.87	21	11	34.37
Iran	23	4.93	15	8	34.78
USA	17	3.65	12	5	29.41
Mexico	15	3.22	10	5	33.33
Thailand	15	3.22	11	4	26.66
Sweden	14	3.00	6	8	57.14
Chile	12	2.57	11	1	8.33
Bangladesh	11	2.36	6	5	45.45
Malaysia	11	2.36	4	7	63.63
Norway	11	2.36	4	7	63.63
Italy	10	2.16	6	4	40.00
Indonesia	9	1.93	6	3	33.33
Pakistan	9	1.93	4	5	55.55
Belgium	8	1.72	4	4	50.00
Australia	6	1.29	2	4	66.66
Japan	6	1.28	0	6	100.00

SCP: single country publications, MCP: multiple country publications.

**Table 2:** The top-cited documents on SC-related documents in aquaculture (2015–2025). SC: *Saccharomyces cerevisiae*.

Rank	Paper	C1	C2	Citations
1	Dawood MAO, 2016, Aquaculture	372	37.20	[36]
2	Oberbeckmann S, 2016, PLoS One	372	37.20	[37]
3	Hai NV, 2015, J APPL Microbiol	348	31.63	[38]
4	Carbone D, 2016, Fish Shellfish Immunol	276	27.60	[39]
5	Kabeya N, 2018, Sci Adv.	178	22.25	[40]
6	Hai NV, 2015, Fish Shellfish Immunol	175	15.91	[41]
7	Sharif M, 2021, Aquaculture	172	34.40	[42]
8	Jannathulla R, 2019, Aquac Res	169	24.14	[43]
9	Dawood MAO, 2016, Fish & Shellfish Immunol	166	16.60	[44]
10	Standen BT, 2016, Fish & Shellfish Immunol	166	16.60	[45]
11	Rohani MF, 2022, Fish & Shellfish Immunol	157	39.25	[46]
12	Overland M, 2017, J Sci Food Agric.	140	15.55	[47]
13	Iwashita MKP, 2015, Fish Shellfish Immunol	124	11.27	[48]
14	Huyben D, 2018, J APPL Microbiol	114	14.25	[49]
15	Dawood MAO, 2017, Aquac Nutr	113	12.55	[50]
16	Abdel-Tawwab M, 2018, Fish & Shellfish Immunol	109	13.62	[51]
17	Dawood MAO, 2016, Fish Shellfish Immunol	104	10.40	[52]
18	Zhou P, 2015, Appl. Microbiol Biotechnol	101	09.18	[53]
19	Wang YC, 2019, Fish & Shellfish Immunol	93	13.28	[54]
20	Lin HL, 2017, Fish &Shellfish Immunol	90	10.00	[55]

“C1”: Total number of citations, “C2”: citations/year.

## Data Availability

Data supporting this study’s findings are available from the corresponding author upon request.
